# SLAM-Based Self-Calibration of a Binocular Stereo Vision Rig in Real-Time

**DOI:** 10.3390/s20030621

**Published:** 2020-01-22

**Authors:** Hesheng Yin, Zhe Ma, Ming Zhong, Kuan Wu, Yuteng Wei, Junlong Guo, Bo Huang

**Affiliations:** 1 State Key Laboratory of Robotics and System, Harbin Institute of Technology, Harbin 150001, China; 18b908038@stu.hit.edu.cn; 2 Industrial Research Institute of Robotics and Intelligent Equipment, Harbin Institute of Technology, Weihai 264209, China; 171320515@stu.hit.edu.cn (Z.M.); zhongming@hit.edu.cn (M.Z.); junlongg@hit.edu.cn (J.G.); 3 Sphyrna Technology Company, Beijing 100096, China; kwu@shuangjisha.com (K.W.); ytwei@shuangjisha.com (Y.W.)

**Keywords:** self-calibration, binocular stereo vision rig, extrinsic parameter, SLAM

## Abstract

The calibration problem of binocular stereo vision rig is critical for its practical application. However, most existing calibration methods are based on manual off-line algorithms for specific reference targets or patterns. In this paper, we propose a novel simultaneous localization and mapping (SLAM)-based self-calibration method designed to achieve real-time, automatic and accurate calibration of the binocular stereo vision (BSV) rig’s extrinsic parameters in a short period without auxiliary equipment and special calibration markers, assuming the intrinsic parameters of the left and right cameras are known in advance. The main contribution of this paper is to use the SLAM algorithm as our main tool for the calibration method. The method mainly consists of two parts: SLAM-based construction of 3D scene point map and extrinsic parameter calibration. In the first part, the SLAM mainly constructs a 3D feature point map of the natural environment, which is used as a calibration area map. To improve the efficiency of calibration, a lightweight, real-time visual SLAM is built. In the second part, extrinsic parameters are calibrated through the 3D scene point map created by the SLAM. Ultimately, field experiments are performed to evaluate the feasibility, repeatability, and efficiency of our self-calibration method. The experimental data shows that the average absolute error of the Euler angles and translation vectors obtained by our method relative to the reference values obtained by Zhang’s calibration method does not exceed 0.5˚ and 2 mm, respectively. The distribution range of the most widely spread parameter in Euler angles is less than 0.2˚ while that in translation vectors does not exceed 2.15 mm. Under the general texture scene and the normal driving speed of the mobile robot, the calibration time can be generally maintained within 10 s. The above results prove that our proposed method is reliable and has practical value.

## 1. Introduction

The practical application of binocular stereo vision (BSV) rig in the market of unmanned vehicles and mobile robots as sensing equipment has been greatly challenged [1,2]. There is still room for further improvement in the practicability and durability of the BSV rig. The lack of practicality is mainly reflected in its structural form. The positions of two cameras for most existing BSV rigs are relatively fixed, which means the operators can hardly adjust the baseline. This form makes it difficult for binocular vision to be installed in different sized spaces and cannot satisfy the measurement and sensing range requirements when the robot faces different scale scenes. The drawback of durability is mainly reflected in the fact that BSV rig is often deformed due to temperature, vibration, etc., resulting in changes in the parameters calibrated at the factory. For example, to keep the calibration parameters from deviations, the structure of the rig is usually made of special materials and needs to be firmly connected with robots or cars. As a result, the university research team, autopilot company, and automotive aftermarket industry have to recalibrate the BSV rig frequently for the result accuracy. It is well known that calibration and recalibration of the rig have always been a burden for each user, which requires expertise, specialized equipment and many hours of work [3]. Consequently, it is necessary to study an automatic, robust, real-time calibration algorithm without prior information of the environment, human supervision and auxiliary equipment.

With the popular application of BSV rig in computer vision [4], many methods have been proposed to calibrate them. Most existing calibration methods are based on manual off-line calibration algorithms for specific reference targets or patterns, such as the traditional calibration methods, planar template methods [5], 3D-object-based calibration methods [6]. Typical algorithms of traditional calibration methods include the direct linear transformation algorithm (DLT) [7], the nonlinear optimization algorithm [8], and the Tsai’s radial alignment constraint algorithm (RAC) [9]. Deng et al. [10] proposed a relational model for camera calibration, which takes into account the camera’s geometric parameters and lens distortion effects. The combination of differential evolution and particle swarm optimization algorithm can effectively calibrate camera parameters. Batista et al. [11] used monoplane calibration points to achieve camera calibration. To avoid the singularity obtained by the calibration equation when using monoplane calibration points, a multi-step procedure combined with a nonlinear optimization iterative process is used to solve these parameters and improve the precision. Zhuang et al. [12] used an RAC method to calibrate the camera through a 2D planar calibration plate parallel to the camera plane. This method combines the advantages of traditional linear and non-linear optimization algorithms to simplify the parameter solving process, so the calibration results are relatively accurate. The traditional calibration method can accurately calibrate the camera parameters utilizing precisely fabricated planar or stereo targets [13]. However, the calibration algorithms and procedures are complex and time-consuming, so they are suitable for where camera parameters are not changed often.

To avoid the shortcomings of traditional methods, the planar template method has been extensively studied. Zhang [14] proposed to calibrate the camera with a checkerboard, which is widely used. The camera is required to view the displayed checkerboard pattern in several different directions. Yu et al. [15] proposed a robust recognition method of checkerboard pattern detection for camera calibration, which is based on Zhang’s method. Chen et al. [16] described a novel camera calibration method that used only a single image of two coplanar circles with arbitrary radii to estimate the camera’s extrinsic parameters and focal length. Kumar et al. [17] proposed a technique for camera calibration using a planar mirror. By allowing all cameras to see multiple fields of view through the mirror, it is possible to overcome the need for all cameras to see a common calibration object directly.

The 3D target-based method makes the calibration procedure more simplified by placing the target in the camera field of view once to obtain calibration parameters. Su et al. [18] used a spherical calibration object as a specialized reference pattern to calibrates the geometric parameters between any number of cameras on the network. Zhen et al. [19] proposed a method for calibrating extrinsic parameters of BSV rig based on double-ball targets. A target consisting of two identical spheres fixed at a known distance is freely placed in different positions and orientations. With the aid of markers, the calibration precision is high, and the calibration process is simpler, but the calibration method is still not flexible enough. Since it is difficult to machine 3D targets and keep the calibration images of all feature points at the same sharpness, therefore, their practical application is limited.

Unlike the above-mentioned calibration methods, the self-calibration method only requires a constraint from the image sequence without any special reference objects or patterns designed in advance, which may allow online calibration of camera parameters in real-time. Luong et al. [20] proposed earlier to calibrate the extrinsic parameters of BSV rig by point-matching methods in general unknown situations. Using the SIFT [21] feature point correspondence and bundle adjustment (BA) algorithm, Tang et al. [22] proposed a local-global hybrid iterative optimization method for calibration of BSV rig. Wang et al. [23] proposed a self-calibration method using sea surface images, which can estimate the rotation matrix of a BSV rig with a wide baseline. Boudine et al. [24] proposed a self-calibration technology for BSV rig with variable intrinsic parameters, which is based on the relationship between two matching terms (i.e., the projection of two points representing the vertices of the triangle isosceles rectangular triangle) and the absolute cone image. Ji et al. [25] introduced a calibration model for the multiple fisheye camera rig which combines a generic polynomial and an equidistance projection model to achieve high-accuracy calibration from the fisheye camera to an equivalent ideal frame camera. Wang et al. [26] proposed a self-calibration method that uses a single feature point and converts the pitch and yaw imaging model into a quadratic equation of the pitch tangent value. However, the calibration process for the above work still requires complex user intervention. Some methods require the user to manually specify the point correspondence between the two camera views of BSV rig, which seems inconvenient in practice.

So far, we have found the work of Carrera et al. [27] is closer to ours. They used the improved monocular vision Mono-SLAM algorithm to process the video sequence of each camera separately, then robustly match and fuse the maps obtained by each camera based on the corresponding SURF [28] features. Finally, the extrinsic parameters between multiple cameras can be calibrated through matching the global map with the SURF features extracted from cameras. However, this method does not provide real-scale information on the surrounding environment, and its calibration accuracy and efficiency have yet to be further verified. Heng et al. [29] also used the visual SLAM-based self-calibration method to calibrate the BSV rig extrinsic parameters fixed on the aircraft and provide the scale information through the three-axis gyroscope. Heng et al. [30] further applied a similar idea and method to the calibration of the BSV rig mounted on car platforms and provided the scale information through the odometer to make the algorithm applicable to large scenes. However, the calibration efficiency of this method limits its practical application.

Based on the summary analysis of related research fields, there are still some problems in the calibration work of the BSV rig. The calibration process employing the auxiliary device is complicated and inconvenient for the users to personally operate. The self-calibration method is still in the research and exploration stage, and its calibration efficiency and accuracy have yet to be further verified. To the best of our knowledge, there is no existing self-calibration method that can be practically applied to the calibration of BSV rig in a wide range and achieve commercial value.

In this paper, we propose a novel SLAM-based self-calibration method for the BSV rig. The purpose is to achieve real-time, automatic and accurate calibration of the BSV rig’s extrinsic parameters in a short period. The intrinsic parameters of the left and right cameras are estimated in advance using Zhang’s method. And as far as possible, this calibration method can be used for large-scale practical applications on arbitrary BSV rigs, such as a car or mobile robot.

The main contributions of our work are reflected in the following aspects:

1. Our proposed SLAM-based self-calibration method can estimate the extrinsic parameters of the BSV rig without auxiliary equipment and special calibration markers.

2. Once the baseline is determined, a fully automatic calibration process can be implemented in real-time during the normal driving of the platform, without complex requirements for rig movement and prior information about the environment.

3. In the experimental part, the proposed self-calibration method was applied to the mobile platform where the BSV module is installed. Our method can accurately calibrate the BSV rig in a short period. As far as we know, there are few existing methods to perform the self-calibration of BSV rig in such high efficiency, thereby achieving the effect of practical application.

The rest of the paper is organized as follows: Section 2 describes our SLAM-based self-calibration pipeline. Section 3 discusses the SLAM-based construction pipeline of a 3D scene point map that used to generate a calibration area map. Section 4 introduces the extrinsic parameter calibration algorithm of BSV rig. Section 5 explains the experimental setup and verification results. Conclusions and future work are presented in Section 6.

## 2. SLAM-Based Self-Calibration Pipeline

This section describes our SLAM-based self-calibration pipeline as a whole, which can estimate the extrinsic parameters of the BSV rig. Here, the main contents of the pipeline are briefly summarized and described in the following sections.

**Figure 1:**
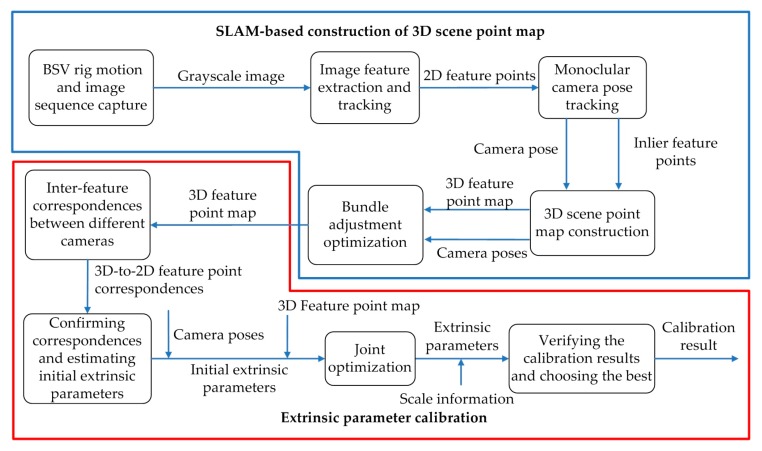
Simultaneous localization and mapping (SLAM)-based self-calibration pipeline which estimates the extrinsic parameter of the binocular stereo vision (BSV) rig.

Figure 1 shows our SLAM-based self-calibration pipeline. The method is mainly divided into two parts: SLAM-based construction of a 3D feature point map of the natural environment (also called 3D scene point map) and extrinsic parameter calibration. SLAM-based construction of the 3D scene point map is mainly introduced throughout Section 3. To improve the efficiency of calibration, a lightweight, real-time visual SLAM system is built in Section 3.1. The image sequence is captured in the process of BSV rig motion and converted to Grayscale image before being fed to the SLAM process described in Section 3.2. The monocular camera pose tracking step detailed in Section 3.3 estimates camera poses and track inlier feature points extracted from the image. Then, the tracked inlier feature points together with camera poses are used to construct the 3D scene point map of the natural environment described in Section 3.4. The scene point map is composed of a series of 3D feature points, so we also call it a 3D feature point map. To improve the precision of the algorithm, the BA is used to optimize the established 3D feature point map described in Section 3.5.

At that time, an accurate 3D scene point map will be obtained, which can be used as a calibration area. The pipeline then goes into the extrinsic parameter calibration phase detailed in Section 4. The 3D-2D correspondences between the 3D scene points associated with the 2D feature points in the left camera and the 2D feature points in the right camera are obtained through inter-feature correspondences between two binocular cameras described in Section 4.1. Further, the Perspective-n-Point (PnP) method combined with the random sample consensus (RANSAC) algorithm is used to confirm correspondences and estimate the initial extrinsic parameters of BSV rig described in Section 4.2. Subsequently, this initial estimation is used together with the camera poses and 3D feature point map by the joint optimization described in Section 4.3 to acquire accurate extrinsic parameters. Finally, the scale information is given in Section 4.4 and the calibration results are verified to select the optimal result.

## 3. SLAM-Based Construction of 3D Scene Point Map 

This section describes our SLAM-based construction of the natural environment that used to produce a map of the calibration area.

### 3.1. Lightweight Monocular Visual SLAM System

**Figure 2:**
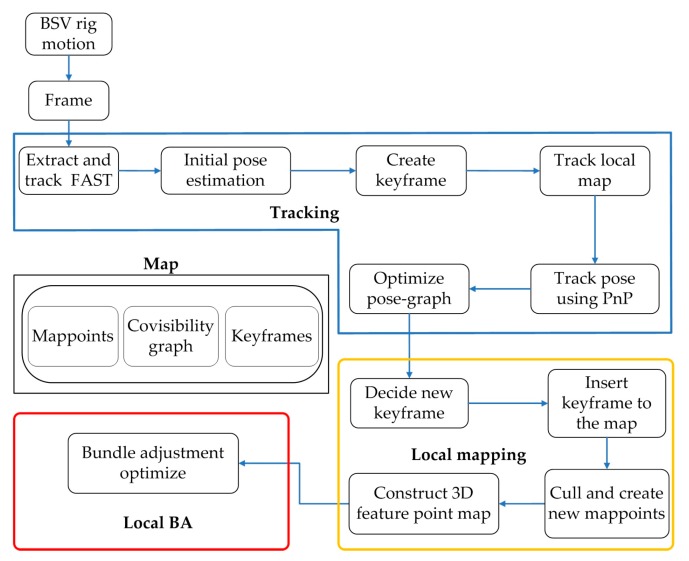
Lightweight monocular visual SLAM system.

Garrigues et al. [31] proposed a features from accelerated segment test (FAST)-based optical flow method whose efficiency and performance are better compared to the feature corresponding methods based on slow corner points such as Harris [32], SIFT, and SURF. Therefore, the FAST-based optical flow method is chosen as the basic framework of our SLAM algorithm. However, the existing SLAM algorithm framework is more complicated, it is difficult to directly apply to solve our problems and meet the requirements for calibration efficiency. Therefore, a lightweight monocular vision SLAM system is built as shown in Figure 2. The system is already reflected in Figure 1, and the key points in the framework are detailed in the following sections. Therefore, only further supplements to the SLAM system are made here.

The system is mainly composed of four parts: tracking, local mapping, local BA and map. The tracking part locates each frame by extracting and tracking feature points on the image and finds local map features for matching. The local mapping part determines the new keyframes, decides to delete or add new mappoints, and builds a 3D feature point map accordingly. The local BA part manages and optimizes the local map by performing BA. The map part is used to maintain all mappoints, covisibility graph, and keyframes.

The loop-closing detection part like most SLAM algorithms is not performed, because the BSV rig can be calibrated in a shorter path. If the loop-closing detection is added, the place recognition part also needs to be further added, which will increase the complexity of the algorithm.

### 3.2. BSV Rig Motion and Image Sequence Capture

In this step, the sequence of the monocular camera image is captured during camera motion and begins as a SLAM algorithm. To ensure the convenience and practicability, the self-calibration method can be fully automated in real-time during the normal driving of the platform, without complex requirements for rig movement. The movement form of BSV rig is to include as much translation movement as possible along the opposite direction of the camera’s optical axis, and this kind of movement is also a common action on mobile platforms. Here, it is assumed that the two monocular cameras of the BSV rig has at most a minimum overlapping field of view. For example, the calibration can be performed during the normal forward or backward movement of the car or mobile robot. Aircraft operating in tight spaces can also be calibrated during vertical movement. Of course, this form of movement is ideal, which can ensure the precision and efficiency of the calibration to the greatest extent. This point is analyzed during the initialization of the camera pose tracking in Section 3.3. But it is not limited to this way. This is mainly since the calibration is only performed when a keyframe is encountered. At this time, the local mapping part has already saved the feature point map and image feature points required for calibration.

The image sequences are captured by two monocular cameras of identical configuration while ensuring time synchronization. The algorithm does not require any prior information about the environment, nor does it need to set special markers or patterns in the scene. The image sequence should be converted from RGB to Grayscale before being fed to SLAM.

### 3.3. Monocular Camera Pose Tracking

The monocular camera poses and inlier feature points are tracked in the camera pose tracking step. The inlier feature points are required for 3D scene point map construction in Section 3.4.

To decrease the time to track the monocular camera pose, the optical flow method is employed to track the FAST detectors as the feature points. In this paper, optical flow information is used for feature corresponding, mappoint selection, outlier feature points culling and pose estimation. The calculation of partial pixel optical flow is referred to as a sparse optical flow, and the sparse optical flow is represented by a Lucas-Kanade optical flow [33]. The FAST detector is a kind of corner point, which mainly detects the obvious change of local pixel grayscale, and is known for its fast speed [34]. The 16-dimensional grayscale description, block optical flow estimation, and gradient descent search for each FAST corner point are the highlights of its success. Therefore, the LK optical flow method is used to track FAST detectors as feature points in this paper. Besides, this paper adopts the classic corner extraction and optical flow tracking strategies in [31]. However, they do not pay attention to the distribution of corners, but the uniform distribution of corners is intuitively important for us to obtain accurate and fast calibration results.

**Figure 3:**
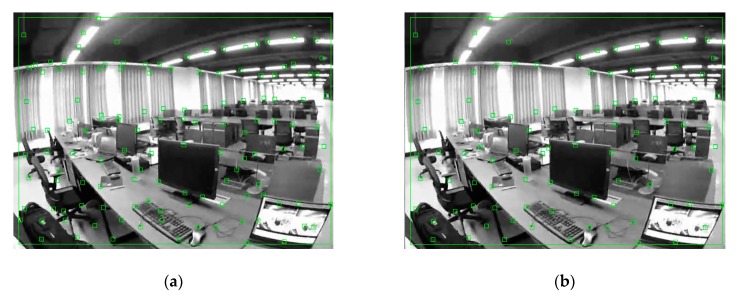
Feature points extraction in the grayscale image: (**a**) Original features from accelerated segment test (FAST) corner points distribution; (**b**) FAST corner points distribution after sparse processing.

The feature points we extracted on the grayscale image are represented by green rectangles in Figure 3a. They are mainly located in areas where the grayscale values are relatively changed, and most of them are located in the corner points. However, they often appear to be "get together" as shown in Figure 3a. That is, the distribution of feature points is too crowded in a certain local area. This problem is easy to cause feature tracking errors, resulting in feature mismatch, which in turn affects camera pose tracking and 3D scene point map construction. Therefore, we added a sparse and uniform distribution strategy for feature points and hope to distribute the corner points as accurately as possible in all areas of the image.

**Figure 4:**
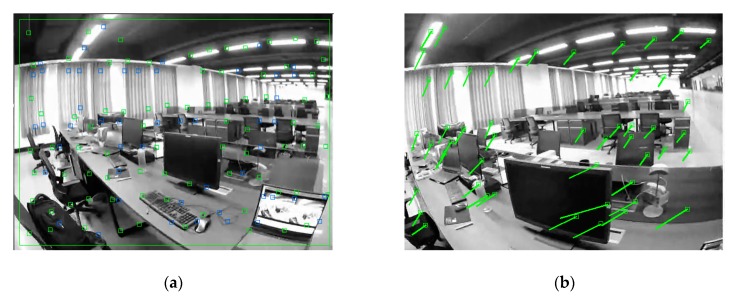
(**a**) FAST corner points distribution managed by the gridded map strategy; (**b**) Feature points tracked by the optical flow method.

In terms of feature points sparseness, the non-maximal suppression method [35] is used to filter local non-maximal points. The effect of the FAST corner distribution after sparse processing is shown in Figure 3b. To improve the feature corresponding performance, a gridded map management strategy similar to the method proposed in [36] is used to maximize the chance of detecting a significant number of corresponding features between the overlapping view of two monocular cameras. The strategy procedure is described as follows: 1. Mesh the feature point map; 2. Count the number of feature points in each grid; 3. Delete the feature points that are too close; 4. If there are no feature points in the grid, select the most significant FAST feature extracted in the region as the new feature point. Thereby, the density under sparseness can be maintained, and the desired number of seed feature points can be ensured for subsequent tracking. Figure 4a shows a feature point map managed by the gridded map, in which a blue rectangle represents an added feature point. The optical flow method is then used to track the feature points processed by the sparse and uniform distribution strategy. The tracking effect is shown in Figure 4b.

**Figure 5:**
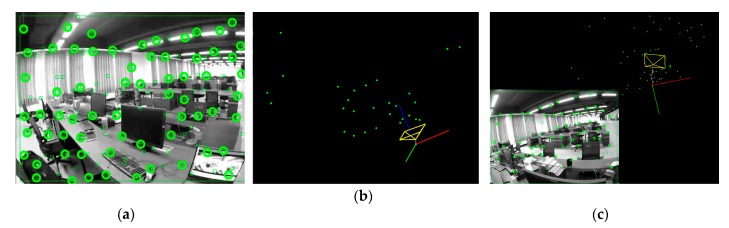
(**a**) Co-vision feature points in the current frame; (**b**) Initial frame pose of the monocular camera; (**c**) Inlier feature points and camera pose tracking.

Before tracking the monocular camera pose, the SLAM needs to be initialized first for tracking. The initialization task includes selecting two suitable monocular frames as the initial frames, estimating the relative pose of the initial frames, creating a local map, and creating a keyframe. According to our usage scenario, the initialization can be divided into the following three stages:

(1) Two initial frames that can be used as the first two frames are selected by feature point corresponding. When selecting the initial frames, it is necessary not only to ensure there are enough corresponding feature point pairs in the current and the last frames but also ensure there are as many co-vision feature points as possible on the two monocular cameras. The specific implementation algorithm is that when the BSV rig moves roughly in the opposite direction of the monocular camera’s optical axis, the camera frame sequence is queried from the current frame in reverse order. When the motion of more than *N* ∈ **Z** corresponding feature point pairs is greater than *M* ∈ **Z** pixels, it is considered that the pixel moving distance is large enough. The frame that is queried in reverse order at this time is taken as a reference frame. The current frame and the reference frame are selected as the initial frames and are used for initialization. The *M* is related to image resolution, and there is a proportional relationship between them.

(2) The co-vision feature points set ***P****_c_* of the current frame and the reference frame is taken out, and the corresponding feature point pairs set ***P***_1_ and ***P***_2_ between the two frames are obtained. The co-vision feature points represented by circles in the current frame are shown in Figure 5a. Combining the Perspective-3-Points [37] with the random sample consensus (RANSAC) [38] algorithm, the essential matrix ***E*** between two frames is calculated. Singular Value Decomposition is then used to calculate the camera motion ***T****_Ini_* between two frames. The reference frame is set to the world frame. The camera pose estimation of the current frame is set to the initial frame pose. As shown in Figure 5b, the world frame is represented by three orthogonal axes (red, green, and blue axes), and the initial camera pose is represented by a yellow cube.

(3) The initial feature point depth is obtained by triangulation, thereby obtaining an initial local map. The resulting local feature points map is represented in Figure 5b by a series of green dots.

After the initialization is completed, the current frame is used as the keyframe, and the initial frame pose and local map are optimized using BA optimization. Our algorithm will perform BA as soon as a new keyframe is obtained, which will be described in detail in Section 3.5. In the camera pose tracking process after obtaining the keyframes, the subsequent frames are corresponded with the local map to calculate the camera poses. The PnP method combined with the RANSAC algorithm is used to estimate camera pose. Here, the Levenberg–Marquardt algorithm [39] is used to minimize the distance between corresponding feature point pairs to calculate the pose of each frame.

However, the RANSAC uses only a few random points to determine the inliers, this method is susceptible to noise. To make the joint optimization more likely converge to the correct solution, the RANSAC solution is used as the initial value, and the camera poses are optimized using the pose-graph method to acquire a globally consistent camera pose estimation. In the pose graph optimization, nodes are used to represent the absolute poses to be optimized, expressed in *c*_0_, …*c_i_*,…*c_m_*. *m* ∈ **Z** is the total number of nodes. An absolute pose *c_i_* describes the transformation from the world frame to the camera frame *i* ∈ **Z**. Edges are used to represent relative pose constraints between two pose nodes. A relative pose constraint $c_{ij}$ between two camera frames *i* and *j* ∈ **Z** is defined as $c_{ij}\;:=\;c_{i}{}^{-1}\cdot \;c_{j}$. Using ε as the set of all edges, the objective function in the optimization can be expressed as:

(1) $$\underset{c}{\min}\sum_{i,j\in \varepsilon }^{}{\Delta c_{ij}}^{T}\Omega_{ij}\Delta c_{ij},$$

where ***C*** is the current set of all camera poses that need to be optimized; $\Delta c_{ij}=\ln(c_{ij}\cdot c_{j}{}^{-1}\cdot c_{i})$ is the relative pose constraint error in the tangent space of SE(3); $\Omega_{ij}$ is an information matrix, which is an inverse matrix of the covariance matrix of relative pose constraint. Rather than using appropriate marginalization to accurately estimate this uncertainty, it is better to approximate the information matrix roughly as a diagonal matrix in the way proposed in [40]:

(2) $$\Omega_{ij}=\omega_{ij}\left[\begin{array}{cc} \delta_{t}{}^{2}I_{3\times 3} & 0_{3\times 3}\\ 0_{3\times 3} & \delta_{r}{}^{2}I_{3\times 3} \end{array}\right],$$

where the $\delta_{r}$ and $\delta_{t}$ are the rotational component and the translational component of $\Delta c_{ij}$, respectively. It can be considered the camera pose constraints generated during the optimization are similar in accuracy [41]. Therefore, $\omega_{ij}$ is set to a constant $\omega_{ij}$ = 1.

Figure 5c shows the results of inlier feature points and camera poses tracking. The brown line connected to the origins from the world frame to the current frame represents the tracked camera poses, and the yellow dot on the brown line represents the keyframe poses.

### 3.4. 3D Scene Point Map Construction

This step is used to construct the 3D feature point map of the natural environment which is used as a calibration area. The monocular camera poses and inlier feature points are input in this step, the output is 3D scene point map of the tracked inlier feature points in the keyframe. Note that the new keyframes are not determined due to the reduction of tracked feature points in consecutive frames. On the contrary, the new keyframe in the local map must have as many common views with other keyframes as possible to increase the number of 3D scene points required for calibration.

**Figure 6:**
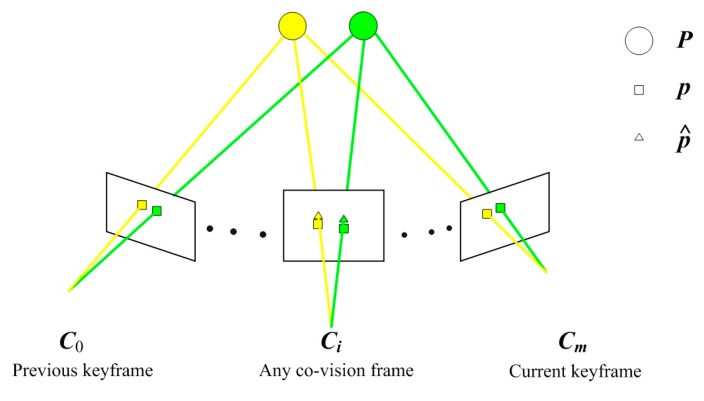
Schematic diagram of 3D feature points map construction.

Figure 6 is a schematic diagram of our 3D feature point map construction. The Vector ***C*** represents the camera pose set of all co-view frames between the current keyframe and the previous keyframe; The vector ***P*** represents a 3D scene point map, which is represented by circles; The vector ***p*** represents the set of 2D feature points extracted and tracked in the image, and is represented by rectangles; The vector $\hat{p}$ represents the set of 2D points projected from the local map onto the co-view frames, and is represented by triangles; The connection between the 3D feature points and the 2D feature points indicates the co-view relationship, and the yellow and green lines are used to distinguish them.

(3) $$\begin{array}{l} C\;=\;\{c_{0},\ldots c_{i},\ldots c_{m}\},\\ P\;=\;\{P_{0},\ldots P_{j},\ldots P_{n}\},\\ p\;=\;\{p00,\ldots ,p0n,\ldots p_{i}{}_{0},\ldots p_{ij},\ldots ,p_{in},\ldots p_{m}{}_{0},\ldots ,p_{mn}\},\\ \hat{p}\;=\;\{\hat{p}00,\ldots ,\hat{p}0n,\ldots {\hat{p}}_{i}{}_{0},\ldots {\hat{p}}_{ij},\ldots ,{\hat{p}}_{in},\ldots {\hat{p}}_{m}{}_{0},\ldots ,{\hat{p}}_{mn}\}, \end{array}$$

where $c_{i}$ is the absolute pose of co-view frame *i* ∈ **Z**, *m* is the total number of co-view frames; ***P****_j_* is the coordinate of 3D feature point *j* ∈ **Z** in the world frame, *n* is the total number of 3D feature points; ***p****_ij_* is the observed image coordinate corresponding to 3D feature point *j* in the co-view frame *i*; ${\hat{p}}_{ij}$ is the estimated image coordinate of 3D feature point *j* projected in co-view frame *i*.

(4) $$\left\{ \begin{array}{l} \;\;\frac{n{\left\|p_{ij}-{\hat{p}}_{ij}\right\|}^{2}}{\sum_{j=1}^{n}{\left\|p_{ij}-{\hat{p}}_{ij}\right\|}^{2}}\leq T\;\begin{array}{c} \mathrm{Yes}\;P_{j}\text{ is a 3D feature point that can be retained;} \end{array}\\ \;\;\frac{n{\left\|p_{ij}-{\hat{p}}_{ij}\right\|}^{2}}{\sum_{j=1}^{n}{\left\|p_{ij}-{\hat{p}}_{ij}\right\|}^{2}}>T\;\begin{array}{c} \mathrm{No}\;P_{j}\text{ is a 3D feature point to be deleted.} \end{array} \end{array}\right.$$

When the SLAM creates a new keyframe, triangulation is used to build a local map. To reduce the inaccurate map caused by false tracking and noise, 3D feature points are projected onto any co-vision frame. When the reprojection error exceeds *T* ∈ **Z** times average error, the 3D feature point ***P****_j_* is considered to be mismatched and deleted. This can improve the calibration precision to a certain extent.

### 3.5. Bundle Adjustment of Camera Pose and 3D Scene Point Map

The camera pose obtained in Section 3.3 and the 3D feature point map obtained in Section 3.4 are jointly optimized in this step for 3D scene point map refinement. The cost function of this optimization can be regarded as a nonlinear least-squares problem. In Formula (5), the cost function corresponds to the reprojection errors of the feature observations across all keyframes:

(5) $$\underset{c_{i},P_{j}}{\min}\sum_{i=1}^{m}\sum_{j=1}^{n}\rho \left({\left\|p_{ij}-\pi \left(c_{i},P_{j}\right)\right\|}^{2}\right),$$

where $c_{i}$ is the absolute pose of keyframe *i* ∈ **Z**, *m* is the total number of keyframes; ***P****_j_* is the coordinate of 3D feature point *j* ∈ **Z** in the world frame, *n* is the total number of 3D feature points; ***p****_ij_* is the observed image coordinate corresponding to 3D feature point *j* in the keyframe *i*; π is a standard camera projection function that predicts the image coordinates of the 3D feature point *j* given the camera’s intrinsic parameters and the estimated camera pose $c_{i}$; ρ is a robust kernel used for reducing the effects of outliers. The Huber kernel [42] is used here.

## 4. Extrinsic Parameter Calibration

This section describes how to calibrate the extrinsic parameters through the 3D scene point map created by the SLAM system.

### 4.1. Inter-Feature Correspondences between Different Cameras

In this step, the feature points in the overlapping fields of two monocular cameras are matched to obtain the 2D-2D feature correspondences.

Whenever the 3D scene point map is constructed by the left camera in Section 3.4, the optical flow method is used to perform feature correspondences on the left and right cameras. Compared with the use of descriptors for matching, the method based on optical flow tracking can obtain the feature correspondences faster. However, using this method will return some mismatched points.

To ensure the accuracy of the inter-feature correspondences between different cameras, the cross-correlation verification algorithm based on grayscale similarity is used to filter the feature correspondences. Assume the *i*-th (*i* ∈ **Z**) 2D-2D feature correspondence in the left and right images is recorded as $\left(p_{i}^{l},{\;p}_{i}^{r}\right)$. First, a search window block *K_w_*_× *w*_ with the size of *w*^2^ (*w* ∈ Z) pixels is defined with the seed point $p_{i}^{l}$ as the center in the left image. In the right image, a search window block with the same size as *K_w_*_× *w*_ is also defined with the candidate point ${\;p}_{i}^{r}$ as the center. Then, at the same starting point position, the grayscale sequences of the pixels in the window block are taken in the same order. These two sequences are recorded as $G_{l}(i)$ and $G_{r}(i)$, where *i* = 0, 1, 2 … *w*^2^−1. Based on these sequences, the cross-correlation coefficient in the two window blocks is calculated:

(6) $$Corr(G_{l}(i),\;G_{r}(i))=\frac{\sum_{i=0}^{w^{2}-1}\left[(G_{l}(i)-m_{l})*(G_{r}(i)-m_{r})\right]}{\sqrt{\sum_{i=0}^{w^{2}-1}{\left(G_{l}(i)-m_{l}\right)}^{2}}\sqrt{\sum_{i=0}^{w^{2}-1}{\left(G_{r}(i)-m_{r}\right)}^{2}}},$$

where $m_{l}$ and $m_{r}$ are the arithmetic mean of the sequence $G_{l}(i)$ and $G_{r}(i)$, respectively.

**Figure 7:**
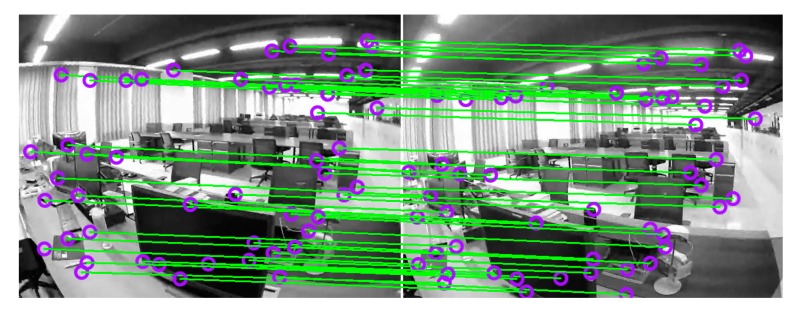
Feature correspondences between the left and right binocular cameras.

The grayscale similarity of the feature correspondence is characterized by the cross-correlation coefficient. When the coefficient exceeds a threshold defined empirically, the gray sequence around the seed point and the candidate point should be highly cross-correlated. Now, the feature correspondence of the optical flow tracking is accurate. Otherwise, the feature correspondence is deleted as a mismatch. Figure 7 shows the result of feature correspondences between the left and right binocular cameras.

According to the 3D scene points associated with the 2D feature points in the left camera, the 3D-2D correspondences between the 3D scene points and the 2D feature points in the right camera are further obtained.

### 4.2. Confirming Correspondences and Estimating Initial Extrinsic Parameters

The goal of this step is to determine which feature correspondences between the two cameras are geometrically consistent and calculate an initial relative transformation between them.

The 3D-2D feature correspondences between the 3D scene points and the 2D feature points in the right camera have been found in the previous section. The RANSAC algorithm is still used to eliminate the outliers and the PnP method is used to solve the initial relative transformation:

(7) $$sp_{i}^{r}=K_{r}T_{l}^{r}P_{i},$$

where the *s* ∈ **R**^+^ is a local scale. Kr is the intrinsic parameter matrix of the right camera. ***P**_i_* is the 3D scene point in the world frame corresponding to the 2D feature point $p_{i}^{r}$. $T_{l}^{r}\in {\mathbb{R}}^{4\times 4}$ is the relative transformation matrix denoted by the Lie group of euclidean transformation [43], and its specific form is as follows:

(8) $$T_{l}^{r}=\left[\begin{array}{cc} R_{l}^{r} & t_{l}^{r}\\ \;0_{1\times 3} & 1 \end{array}\right],$$

where $R_{l}^{r}\in {\mathbb{R}}^{3\times 3}$ is the rotation transformation matrix between two cameras; $t_{l}^{r}\in {\mathbb{R}}^{3}$ is the translation vector.

In the present study, three Euler angles [44] (represented *r_x_*, *r_y_*, and *r_z_*, respectively) about the *X*-axis, *Y*-axis, and *Z*-axis are used to represent the rotation transformation matrix [26], as shown in Formula (9).

(9) $$R_{l}^{r}=R_{z}R_{x}R_{y},$$

where $R_{x}$ = $\left[\begin{array}{ccc} 1 & 0 & 0\\ 0 & \cos(rx) & \sin(rx)\\ 0 & -\sin(rx) & \cos(rx) \end{array}\right]$, $R_{y}$ = $\left[\begin{array}{ccc} \cos(ry) & 0 & -\sin(ry)\\ 0 & 1 & 0\\ \sin(ry) & 0 & \cos(ry) \end{array}\right]$, $R_{z}$ = $\left[\begin{array}{ccc} \cos(rz) & \sin(rz) & 0\\ -\sin(rz) & \cos(rz) & 0\\ 0 & 0 & 1 \end{array}\right]$.

The reason why the Euler angles are used to further express the extrinsic parameters here is the Euler angles provide an intuitive way to describe rotation, which is convenient for evaluating the results obtained in the experimental part.

### 4.3. Joint Optimization

In this step, the monocular camera pose, the 3D scene point map, and the relative transformation between two monocular cameras are jointly optimized to obtain more accurate extrinsic parameters. The cost function is designed to achieve the following goals:

(1). The estimated camera pose of the monocular camera should be accurate.

(2). The 3D feature point map should be accurate.

(3). The relative transformation between the two monocular cameras should be accurate.

In Formula (10), the cost function contains a two-part weighted residual. The first partial residual is the same as Formula (5), corresponding to the reprojection errors of the feature observations for left monocular camera keyframes, while the second part corresponds to those for the right monocular camera frame. At this time, the right monocular camera frame is synchronized to the nearest keyframe in the left camera.

(10) $$\underset{c_{i},P_{j},T_{l}^{r}}{\min}\sum_{i=1}^{m}\sum_{j=1}^{n}w_{1}\rho \left({\left\|p_{ij}-\pi \left(c_{i},P_{j}\right)\right\|}^{2}\right)+\sum_{k=1}^{l}w_{2}\rho \left({\left\|p_{k}-\pi \left(T_{l}^{r},P_{k}\right)\right\|}^{2}\right),$$

where $P_{k}$ is the coordinate of the 3D feature point *k* ∈ **Z** in the world frame, *l* is the total number of 3D feature points observed by the left and right cameras; $p_{k}$ is the observed image coordinate in the right image frame corresponding to the 3D feature point $P_{k}$; $w_{1}$ and $w_{2}$ are the weighting coefficients of the two residuals.

### 4.4. Verifying the Calibration Results and Choosing the Best 

This step mainly provides the scale information based on the extrinsic parameters obtained in the previous step and uses this result to perform stereo rectification on the binocular images. Therefore, the optimal calibration result can be selected by the rectified error.

In the absence of the true coordinates of the marker as a reference, the encoder or IMU can be used to provide the scale information. But not all BSV rigs are equipped with the above sensors or sensor acquisition interfaces. To improve the practicability of the calibration algorithm as well as its application to general equipment, based on our BSV module, the baseline length between the left and right cameras can be conveniently measured. This parameter can provide the scale information of camera extrinsic parameters:

(11) $$s=\frac{l_{l}^{r}}{\left\|t_{l}^{r}\right\|},$$

where $l_{l}^{r}$ is the baseline length. According to the scale *s*, the translation vector $t_{l}^{r}$ in a non-metric unit is converted into ${s\cdot t}_{l}^{r}$ in a metric unit.

The method of stereo epipolar rectification [45] is used to select the optimal calibration result. When using extrinsic parameters to rectify two images, ideally the image coordinates of the feature points corresponding to the same 3D scene point should be on the same polar line. However, due to the imprecision of calibration, there may be some errors in the stereo rectification. According to this characteristic, the errors of stereo rectification are used as a criterion for judging whether the calibration is sufficiently accurate.

At present, the rectification algorithms are mainly divided into two categories, one is calibrated stereo rectification represented by Bouguet algorithm [45], and the other is non-calibrated stereo rectification represented by Hartley algorithm [46]. When the transformation matrix $T_{l}^{r}$ between two cameras is obtained in Section 4.3, the rotation transformation matrix of epipolar rectification can be obtained using the Bouguet method, as shown in Formula (12):

(12) $$\begin{array}{l} R_{l}=R_{rect}{R_{l}^{r}}^{\frac{1}{2}},\\ R_{r}=R_{rect}{R_{l}^{r}}^{-\frac{1}{2}}, \end{array}$$

where $R_{rect}$ is the transformation matrix that makes the line of the camera’s optical center parallel to the image plane. The method of constructing this matrix is completed by the translation vector $t_{l}^{r}$ [45]. $R_{l}$ and $R_{r}$ are the rotation transformation matrices of the left and right cameras before and after rectification, respectively.

Suppose the 2D feature point coordinates on the left and right images corresponding to the same 3D scene point $P_{i}\;=\;{\left[XiYiZi\right]}^{T}$ in the left camera frame are $p_{i}^{l}$ =${\left[u_{i}^{l}\;v_{i}^{l}\right]}^{T}$ and $p_{i}^{r}$ = ${\left[u_{i}^{r}\;v_{i}^{r}\right]}^{T}$. According to the obtained extrinsic parameters, the 3D scene point in the right camera frame can be expressed as:

(13) $$P_{i\;}^{’}=\;{\left[X_{i}^{’}Y_{i}^{’}Z_{i}^{’}\right]}^{T}=\;R_{l}^{r}P_{i}\;+t_{l}^{r}.$$

According to the pinhole camera model, the normalized coordinates of the feature points $p_{i}^{l}$ can be obtained as:

(14) $$\tilde{p_{i}^{l}}=\left[\begin{array}{l} \tilde{u_{i}^{l}}\\ \tilde{v_{i}^{l}} \end{array}\right]=\left[\begin{array}{l} Xi/Yi\\ Yi/Zi \end{array}\right],\tilde{\;p_{i}^{r}}=\left[\begin{array}{l} \tilde{u_{i}^{r}}\\ \tilde{v_{i}^{r}} \end{array}\right]=\left[\begin{array}{l} X_{i}^{’}/Y_{i}^{’}\\ Y_{i}^{’}/Z_{i}^{’} \end{array}\right].$$

The method of distortion rectification [47] is used to remap the positions of the feature points $\tilde{p_{i}^{l}}$ and $\tilde{p_{i}^{r}}$, and the undistorted expression can be written as:

(15) $$\begin{array}{l} U\left(\tilde{p_{i}^{l}}\right)=\tilde{p_{i}^{l}}\cdot (1+k_{l1}r_{l}{}^{2}+k_{l2}r_{l}{}^{4}+k_{l3}r_{l}{}^{6})+\delta \left(\tilde{p_{i}^{l}}\right),\\ U\left(\tilde{p_{i}^{r}}\right)=\tilde{p_{i}^{r}}\cdot (1+k_{r1}r_{r}{}^{2}+k_{r2}r_{r}{}^{4}+k_{r3}r_{r}{}^{6})+\delta \left(\tilde{p_{i}^{r}}\right), \end{array}$$

where $k_{l1}$, $k_{l2}$, $k_{l3}$,$p_{l1}$, $p_{l2}$ are the distortion coefficients of the left camera; $k_{r1}$, $k_{r2}$,$k_{r3}$, $p_{r1}$, $p_{r2}$ are the distortion coefficients of the right camera; ${r_{l}}^{2}={\tilde{u_{i}^{l}}}^{2}+{\tilde{v_{i}^{l}}}^{2}$,${r_{r}}^{2}={\tilde{u_{i}^{r}}}^{2}+{\tilde{v_{i}^{r}}}^{2}$; $\delta \left(\tilde{p_{i}^{l}}\right)$ and $\delta \left(\tilde{p_{i}^{r}}\right)$ both refer to the tangential distortion vector and are expressed as:

(16) $$\delta \left(\tilde{p_{i}^{l}}\right)=\left[\begin{array}{l} 2p_{l1}\tilde{u_{i}^{l}}\tilde{v_{i}^{l}}+p_{l2}\left({r_{l}}^{2}+2{\tilde{u_{i}^{l}}}^{2}\right)\\ 2p_{l2}\tilde{u_{i}^{l}}\tilde{v_{i}^{l}}+p_{l1}\left({r_{l}}^{2}+2{\tilde{v_{i}^{l}}}^{2}\right) \end{array}\right],\;\delta \left(\tilde{p_{i}^{r}}\right)=\left[\begin{array}{l} 2p_{r1}\tilde{u_{i}^{r}}\tilde{v_{i}^{r}}+p_{r2}\left({r_{r}}^{2}+2{\tilde{u_{i}^{r}}}^{2}\right)\\ 2p_{r2}\tilde{u_{i}^{r}}\tilde{v_{i}^{r}}+p_{r1}\left({r_{r}}^{2}+2{\tilde{v_{i}^{r}}}^{2}\right) \end{array}\right].$$

The stereo epipolar rectification of $\tilde{p_{i}^{r}}$ and $\tilde{p_{i}^{r}}$ can be expressed as:

(17) $$\begin{array}{l} C\left(\tilde{p_{i}^{l}}\right)=RlU\left(\tilde{p_{i}^{l}}\right),\\ C\left(\tilde{p_{i}^{r}}\right)=RrU\left(\tilde{p_{i}^{r}}\right). \end{array}$$

Through the above rectification, the binocular images with coplanar line alignment can be obtained. The rectification error can be expressed as:

(18) $$RectErr(R_{l}^{r},t_{l}^{r})=\frac{1}{N}\sum_{i=1}^{N}\left|C\left(v_{i}^{l}\right)-C\left(v_{i}^{r}\right)\right|.$$

Next, the stereo rectification error is used as the standard for verification of calibration results and to select the optimal calibration result.

(19) $$\left\{ \begin{array}{l} RectErr(R_{l}^{r},t_{l}^{r})\leq T\;\mathrm{YES}\;(R_{l}^{r},t_{l}^{r})\text{ meets the requirements,}\\ RectErr(R_{l}^{r},t_{l}^{r})>T\;\mathrm{NO}\;(R_{l}^{r},t_{l}^{r})\text{ does not meet the requirements,} \end{array}\right.,(R_{l}^{r},t_{l}^{r}),(R_{l}^{r},t_{l}^{r})$$

where *T* is a threshold.

## 5. Experimental Verification

This section describes various experiments to verify the feasibility, repeatability, and efficiency of the calibration parameter estimation by our self-calibration method. The first experiment was to evaluate calibration feasibility. In this experiment, Zhang’s checkerboard method was used to provide ground truth data. Then the results from our proposed self-calibration method were compared against those from Zhang’s method. The second experiment focused on verifying the calibration repeatability of the extrinsic parameters and aimed at comparing experiments under different texture environments. The statistics of the calibration efficiency were also made during the experiment.

### 5.1. Experimental Setup

**Figure 8:**
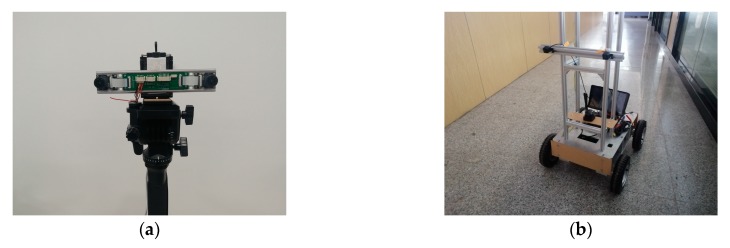
Experiment platform: (**a**) The BSV rig; (**b**) Mobile robot platform.

To verify the proposed self-calibration method, a BSV module was constructed as shown in Figure 8a. The two cameras can be rotated, and their baseline length can be adjusted arbitrarily. The image resolution of the left and right cameras is 640 pixels × 472 pixels and the captured images are transmitted to the computer through a network cable. Table 1 lists the intrinsic parameters of the left and right cameras, including focal length, principal point, and distortion coefficients obtained by Zhang’s method.

Initially, to verify the performance of our calibration algorithm, we performed experiments on a computer, with an Intel Core i7-8750 processor, 8 GB of RAM. To verify the actual application effect of our algorithm in the later period, a mobile robot was used as our experimental platform as shown in Figure 8b. A set of calibration software based on Android system was developed for the convenience of testing. The mobile robot was equipped with a CortexTM-A7 processor, 2 GB of RAM. This processor is currently used in most cars.

### 5.2. Experimental 1-Calibration Feasibility

It is well known that calibration algorithms are difficult to evaluate because it is hard to obtain ground truths of the estimated calibration parameters. Here, direct and indirect methods were used to evaluate the feasibility of the proposed algorithm. In the direct method experiment, the calibration result of Zhang’s method was used as the ground truth data. Zhang’s checkerboard method is widely used in the calibration of the BSV rig with the advantages of low cost, convenience, and high feasibility [26]. Therefore, the calibration results of the proposed method were compared with those produced by Zhang’s method.

**Figure 9:**
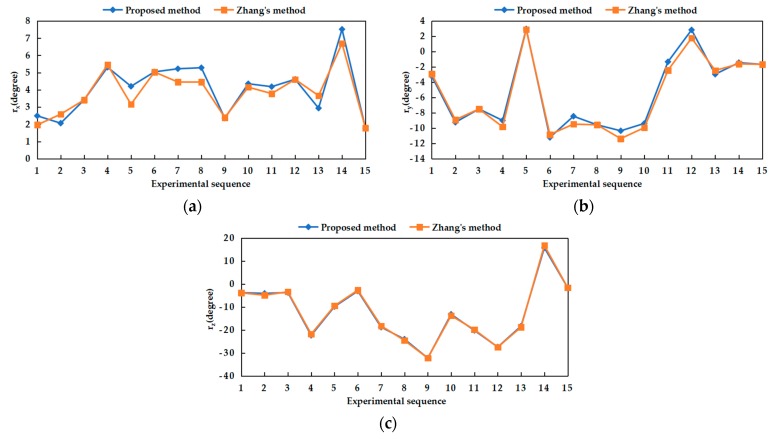
Euler angles between two cameras obtained by our proposed and Zhang’s methods: (**a**) *r_x_* obtained by two methods; (**b**) *r_y_* obtained by two methods; (**c**) *r_z_* obtained by two methods.

**Figure 10:**
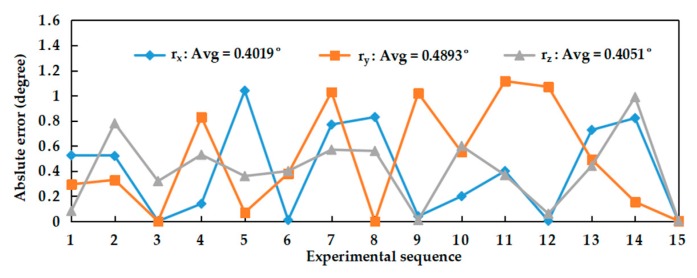
Absolute error of the Euler angles.

Fifteen groups of experiments were performed by adjusting the baseline position and rotation angle between the two cameras of the BSV rig. The three Euler angles transformed from the rotation matrix obtained by the proposed and Zhang’s methods in each experiment were shown in Figure 9, namely *r_x_*, *r_y_*, and *r_z_*. By comparison, it is found that the trends of *r_x_*, *r_y_*, and *r_z_* obtained by two methods were consistent. In Figure 10, the results obtained by Zhang’s method were used as references, and the absolute error of the angles obtained by our proposed method concerning the references was calculated. As shown in Figure 10, the average absolute error of the three angles did not exceed 0.5˚.

**Figure 11:**
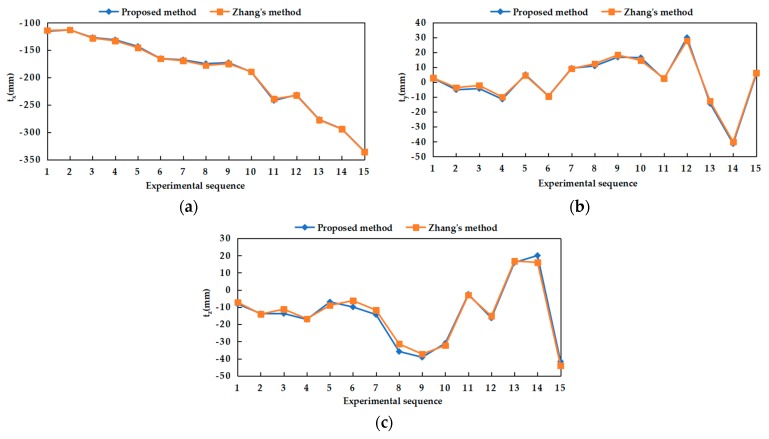
Translation vector between two cameras obtained by our proposed and Zhang’s methods: (**a**) *t_x_* obtained by two methods; (**b**) *t_y_* obtained by two methods; (**c**) *t_z_* obtained by two methods.

**Figure 12:**
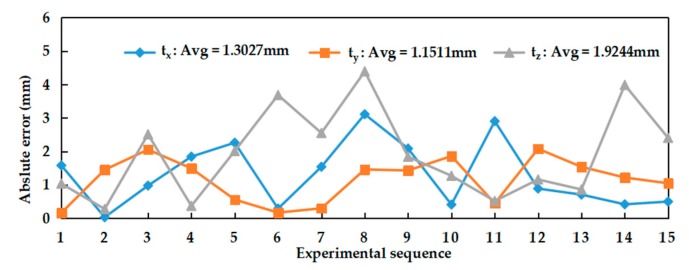
Absolute error of the translation vector.

The results of the translation vector obtained by two methods were shown in Figure 11, namely *t_x_*, *t_y_* and *t_z_*. By comparison, it was also found that the trends of *t_x_*, *t_y_* and *t_z_* obtained by two methods were consistent. The absolute error of the translation vector relative to the reference values is shown in Figure 12. The average absolute error of the translation vector did not exceed 2 mm. Among them, the minimum error of *t_y_* was 1.15 mm.

At the same time, the RMSEs of our and Zhang’s calibration methods were recorded in 15 groups of experiments to evaluate the precision of re-projection. The average RMSE was 0.29 pixel when using Zhang’s method, and 0.51 pixel when using our method. Although Zhang’s method showed higher precision, it can be seen the results obtained by our proposed and Zhang’s method were close, which can prove that our method was reliable.

**Figure 13:**
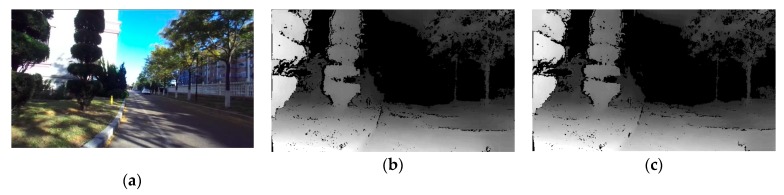
(**a**) The original image; (**b**) The depth map calculated using the parameters estimated by Zhang’s method; (**c**) The right depth map calculated using our estimated parameters.

In the indirect method experiment, the depth image obtained by calibration parameters was used for qualitative evaluation. If the calibration parameters are accurate, the result of the stereo epipolar rectification should be ideal and the depth map formed by the BSV rig will also be dense and accurate. Based on this assumption, the results of the first experimental calibration were selected to perform depth calculations on the original images. Figure 13 showed the depth maps calculated using the parameters estimated by Zhang’s and our method, respectively.

It can be seen from the experimental results that the parameters we estimated would generate a depth map with a uniform density similar to Zhang’s method. This result proved that our estimated parameters have high precision, which can be attributed to the fact: 1. our pipeline filtered and optimized a large number of the tracked feature points; 2. our pipeline verified the results after given the scale parameter and selected the optimal calibration result.

### 5.3. Experimental 2-Calibration Repeatability and Efficiency

This experiment focused on verifying the calibration repeatability of the extrinsic parameters and aimed at comparing experiments under different texture environments. The statistics of the calibration efficiency were also made during this experiment.

**Figure 14:**
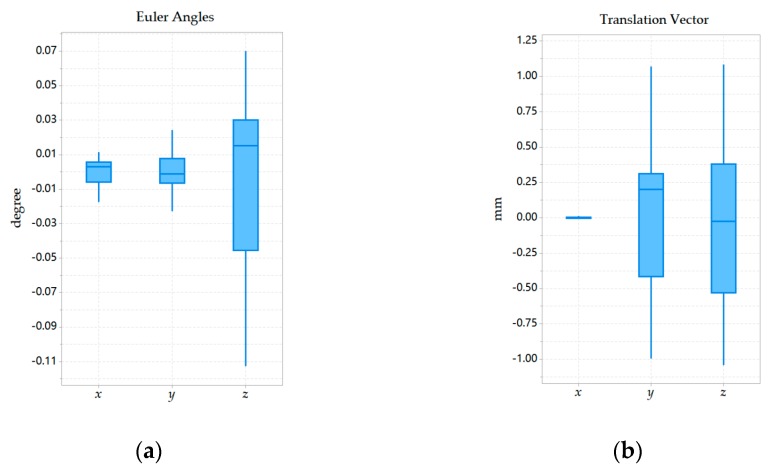
Box plot of the Euler angles and translation vector minus the mean in the first set of experiments: (**a**) Distribution of Euler angles; (**b**) Distribution of translation vector.

**Figure 15:**
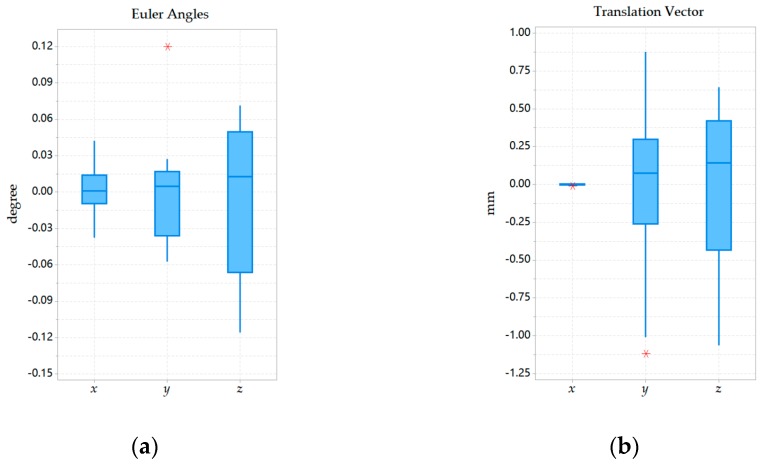
Box plot of the Euler angles and translation vector minus the mean in the second set of experiments: (**a**) Distribution of Euler angles; (**b**) Distribution of translation vector.

The experiment was mainly divided into two groups. The two sets of experiments were performed in the same way under the same camera configuration. In the first set of experiments, a mobile robot was first used to perform 20 experiments under general texture conditions, with approximately 250 feature points extracted. In the second set of experiments, 20 experiments were also performed under weak texture environments, with approximately 80 feature points extracted. The mean and standard deviation of the Euler angles and translation vectors were listed in Table 2. Besides, the distributions of Euler angles and translation vector obtained from the two sets of experiments were recorded in the form of boxplots in Figure 14 and Figure 15. For comparison, the mean was subtracted from the boxplot.

By observing Table 2, it can be found that the largest standard deviation in Euler angles appeared at *r_z_* in the second set of experiments, which is 0.0579˚. The largest standard deviation in the translation vector appeared in *t_y_* of the first set of experiments, which was 0.5850 mm. Therefore, the standard deviation of each parameter in the two sets of experiments was small.

Looking further at Figure 14 and Figure 15, in the first set of experiments, the parameter *r_z_*, which was the most widely distributed parameter in the Euler angles, had a normal interval length of approximately 0.18˚ and the interquartile range of approximately 0.075˚. The most widely distributed parameter in the translation vector was *t_z_*, whose normal interval length was about 2.125 mm and the interquartile range was about 0.9 mm. In the second set of experiments, the parameter *r_z_*, which was the most widely distributed parameter in Euler angles, had a normal interval length of less than 0.2˚ and the interquartile range about 0.1˚. The most widely distributed parameter in the translation vector was *t_z_*, whose normal interval length was less than 2 mm and the interquartile range was about 0.6 mm. Therefore, the distribution of each parameter in the two sets of experiments was concentrated, which showed that our calibration algorithm was repeatable.

At the same time, by comparing the mean and data distribution in the two texture environments, it was found that no major changes occurred, and repeatable results could be obtained. This was mainly due to the re-projection in 3D scene point map construction, all of which were in the range of 0.5–1.0-pixel RMSE. However, through statistical analysis of the calibration time, they were found to be different. The running speed of the mobile robot in both experiments was set to 0.3 m/s. The calibration average time of the first group of experiments was about 7.5 s, while that of the second group was about 2.3 min. Therefore, although the calibration algorithm still maintained certain repeatability under weak texture conditions, it took more time to finish calibration. This was mainly due to our verification and selection of the calibration results because it would take more time to select the required calibration results among the few feature points. However, in practical applications in general scenes, such as outdoor street scenes, the conditions with weak textures are rarely encountered. At the same time, it can be seen that under the general texture scene and the normal driving speed of the robot, the calibration time can be generally maintained within 10 s, which has a higher calibration efficiency. Experimental demonstrations can be found in the supplementary materials.

## 6. Conclusions and Future Work

In this paper, a novel SLAM-based self-calibration method for the BSV rig was proposed. The proposed method was enabled to estimate the extrinsic parameters of the BSV rig without auxiliary equipment and special calibration markers. Further, the calibration process can be fully automated in real-time during the normal driving of the platform, without complex requirements for rig movement and prior information about the environment.

Field experiments were performed to evaluate the feasibility, repeatability of the calibration parameter estimation by our self-calibration method. In terms of feasibility experiments, it can be seen from the experimental data that the results obtained by our proposed and Zhang’s calibration method were close, which could prove that our method was reliable. In the repeatability experiment, 20 experiments performed under general and weak texture environments respectively showed that our calibration method can both obtain relatively compact distribution results. Under the general texture scene and the normal driving speed of the robot, the calibration time can be generally maintained within 10 s, which was more efficient. In short, the experiment results showed that the precision and efficiency of the proposed SLAM-based self-calibration method have reached a relatively high level. This calibration method can be used for large-scale practical applications on arbitrary BSV rigs, such as a car or mobile robot.

However, the calibration method still has shortcomings in the following cases. Firstly, incorrect feature correspondences in a dynamic environment could cause an erroneous 3D scene point map and further lead to unreliable calibration results. Secondly, the baseline length obtained by measurement equipment is adopted as the scale information of the method currently. Although in a more accurate situation, the calibration results can meet the requirements of the advanced driving assistant system. However, calibration precision is still affected by external human factors. Therefore, a suitable method that can automatically calculate the baseline will make the calibration more convenient. In the future, the solution in the dynamic environment and other scale information is the next work we need to focus on.

## Figures and Tables

**Table 1:** Camera intrinsic parameters.

Camera	*f_u_*/Pixels	*f_v_*/Pixels	*u*_0_/Pixels	*v*_0_/Pixels	*k*_1_, *k_2_*, *k*_3_, *p*_1_, *p*_2_
Left	561.86	556.47	318.26	217.06	−0.53, 0.28, 0.003, −0.002, 0.0002
Right	617.91	611.58	312.89	242.26	−0.56, 0.31, 0.004, −0.001, 0.0004

**Table 2:** The mean and standard deviation of Euler angles and translation vectors.

First Set of Experiments		Second Set of Experiments	
Euler Angles (degree)	Translation Vector (mm)	Euler Angles (degree)	Translation Vector (mm)
$\left[\begin{array}{l} 5.2788\\ 2.0636\\ 1.0381 \end{array}\right]\pm \left[\begin{array}{l} 0.0080\\ 0.0111\\ 0.0456 \end{array}\right]$	$\left[\begin{array}{l} -99.9931\\ -0.5070\\ 0.7027 \end{array}\right]\pm \left[\begin{array}{l} 0.0039\\ 0.5850\\ 0.5618 \end{array}\right]$	$\left[\begin{array}{l} 5.2782\\ 2.1244\\ 0.9770 \end{array}\right]\pm \left[\begin{array}{l} 0.0196\\ 0.0418\\ 0.0579 \end{array}\right]$	$\left[\begin{array}{l} -99.9849\\ -0.6226\\ 0.8459 \end{array}\right]\pm \left[\begin{array}{l} 0.0050\\ 0.5448\\ 0.5106 \end{array}\right]$

## Supplementary Materials

The following are available online at https://www.mdpi.com/1424-8220/20/3/621/s1, The experimental demonstration video is available online at https://youtu.be/__wgcoX3jKw.
